# 

**Published:** 1893-01

**Authors:** 


### PAMPHLETS RECEIVED.

We have received from Messrs. P. Blakiston, Son & Co. (1012 Walnut street, Philadelphia) a copy of their Physician's Visiting List for 1893. The fact that the visiting list of the Messrs. Blakiston has reached the forty-second year of its publication is guarantee of its excellence and popularity. This is conveniently arranged for twenty-five patients per week, and contains numerous tables of useful information for ready reference. This makes one of the best visiting lists with which we are acquainted.

Dr. Nicholas Senn, of Chicago, is now preparing a "Syllabus of Lectures on the Practice of Surgery" arranged in conformity with the "American Text Book of Surgery," which will be a valuable aid to all who have this great book. It will be published by W. B. Saunders, Philadelphia.

---

THE Messrs. Macmillan & Co. announce that the recently completed edition of Foster's Text-Book of Physiology in four parts is to be supplemented by the issue of an appendix on "The Chemical Basis of the Animal Body," by A. Sheridan Lea, Sc. D. F. R. S. Dr. Lea is Lecturer on Physiology to the University of Cambridge, England.

---

*Lippincott's Magazine* for January as usual, offers some very choice and interesting reading.

The complete novel of this number, "A Pacific Encounter," by Mary E. Stickney, is a pleasant tale of emotional adventures, matrimonial aspirations and misunderstandings which occur on the good ship "Southern Cross" on the voyage from Panama to San Francisco. It is illustrated.

The *Athletic Series* is continued in an illustrated article on "Foils and Fencing," by Eugene Van Shaick, Captain of the Manhattan Athletic Club.

There are three semi-biographical sketches; one, illustrated by Colin Campbell Cooper, on "A Spanish Painter" (Valesquez); one, by Elizabeth Ballister Bates, on "An Old-Time Philadelphian" (Captain Charles Biddell, 1745-1831); and another, by Alfred Stoddart, on "An Actress and her Art" (Sydney Armstrong). The two latter are accompanied by portraits.

A chapter of Mrs. M. E. Sherwood's reminiscences is headed "In War Time."

H. F. Machuning translates from the French of Emile Bergerat an amusing paper called "A Dictionary Session at the Academy."

M. Crofton in "Men of the Day," describes Emile Zola, Thomas A. Edison and George Du Maurier.

W. S. Walsh gives opinions and extracts from a noted book entitled "Gossip of the Century," and Anne H. Wharton has some remarks on "Recent American Fiction," and especially on Mrs. Atherton's "The Doomswoman."

S. L. Bacon contributes a notable short story (illustrated), "Across Dug Gap."

The poetry of the number is by Ina Lillian Peterson, Carrie Blake Morgan, W. L. Shoemaker, Prof. Chas. G. D. Roberts and William H. Hayne.

